# Plate Teeth

**Published:** 1853-07

**Authors:** J. S. Clark

**Affiliations:** New Orleans


*Plate Teeth.* By J. S. CLARK, D. D. S. New Orleans.

In looking back on the operations of the profession, for the last ten or fifteen years, I am often led to think that we have committed the too common error of "*hobby riding.*" I may be understood as conveying too much meaning by the use of the word *we*, but I speak of the popular practice. Broad plates and narrow plates, broad clasps and narrow clasps, double plates, and single plates, ridge cavity and central cavity plates, have all been tried with commendable zeal; for in this way has the profession advanced, step by step, to a position more worthy the name of DENTAL SURGEONS. But there is one thing that strikes me as too much practiced, which is, that dentists are too apt to apply what they consider the newest and (under some circumstances) best plan for setting plate teeth to *every case.* I am satisfied that there are cases where teeth set on heavy gold wire are the best and most pleasant teeth that can be worn; still it is very seldom I would choose that method, and in the majority of cases such practice is decidedly wrong.

Again, a patient who has a high wedge-shaped alveolar ridge whose posterior termination is not only well defined, but quite prominent, allowing the plate to pass over it cup-shaped, can wear a narrow ridge cavity plate, which would never answer for one whose alveolus is absorbed so as to leave it but little prominence.

I merely mention these simple facts as conducing to a careful study of cases, in which I think the judicious Dentist will come to the conclusion that there are many things thrown by as useless because they happen to be old, and perhaps practiced by the first dentist we ever saw. That thing alone should not condemn any thing really useful. One case in point. The first dentist I ever saw, wielded a hot iron with which he cauterized perhaps indiscriminately. He and his practice are now reckoned as belonging to the "*dark ages.*" But let me ask, have you ever in excavating, say the anterior side of a second bicuspides, (the first being gone,) and finding the fresh nerve exposed in one small point, in the cavity, carefully cauterized it, and filled the tooth, and found, after years, the tooth as apparently healthy and life-like in appearance as its perfect neighbor! The operation is an old one, but in some cases certainly useful, if performed with skill.

In setting plate teeth, I think that the dentist who studies the particular case with the most care, and then makes the best selection of style, drawn from either his own genius or the suggestions of his co-laborers or predecessors without care, except the true interest of his patient, will succeed the best. And he is a happy man, who can with all such assistance and attention, avoid difficulty, in satisfying himself that he has done all that could have been done in replacing those important organs. With these views of a dentist's duty to his patients, I will present my ideas in regard to some peculiar styles of plate teeth, embracing a very unpopular (in the profession) mode of practice.

But first I will say in regard to attachments, etc., in a practice of about fifteen years, I have never filed between the teeth in a single case, and passed a spring through for the attachment of a plate, and I do not do it if the teeth are naturally separated, but prefer fitting a mere bearing on the lingual side only, on each side, and depend on the fit of the plate, across the mouth, to hold the piece generally going back as far for the bearings as possible, that the leverage of the base may bear a large proportion to the leverage of the *tooth.* I have in some instances made bearings entirely on fillings which I regard as the most perfect mode of attachment.

The general fact is that patients wearing a partial set of plate teeth, are on the road to another set, and the mode that injures the remaining teeth the least is certainly the best; and had I, for instance, lost the incisors only, and had perfectly sound molars, I would have a filling put in the lingual side of the first and second molars on each side, and four bearings made against the fillings not touching the teeth at all, thus retaining the plate without injury to the natural teeth.

I have set several artificial pieces in that way, and have been so much pleased, that I will venture to offer it to the profession as worthy of trial. In these cases I have left the filling, say half a line higher in the center, than at the orifice, and in fitting the bearing have made a less but corresponding indentation, so that the plate will spring into place. Great care I think should be taken in finishing the margin so that no receptacle shall be left for foreign deposit.

In backing teeth I think a great point in the attempt at imitation, is lost by the full backing, covering the entire (lingual) surface. Natures teeth are translucent. By a full backing we make them opaque.

In my opinion many attempts at supplying the loss of the bicuspides (only) fail, or become unnecessarily injurious to the neighboring teeth, by putting in teeth with the double cusp. In these cases I prefer the cuspidati.

In the next number, I will present my views in regard to setting plate teeth over fangs, or the practice of their indiscriminate removal.

*Removal of Teeth and Fangs, for artificial pieces.* By J. S. Clark, D. S. S. New Orleans, La.

To be a good follower, or successful practitioner, according to well ascertained principles seems to me to be of far greater importance to the Dental Surgeon than the fame of a discoverer. To me at least, the first is of sufficient importance to claim *my* whole attention, and I trust the day will never come, when we shall so surround ourselves with so many new and brilliant improvements, that we shall forget that a HUNTER, a KOEKER, and a HAYDEN lived, toiled, and died, leaving rich legacies to our noble profession.

I hold it as a sacred duty to cherish their memories, and imitate their devotion. From the practice and the writings of such pioneers, we draw what we call correct rules of practice. Rules of well demonstrated utility. But our profession is not called upon to stop where they stopped, to close our eyes with fear when they doubted, and blindly to worship their operations as *the practice*. Shame on us, if we starting where they left off (as to principles,) make no advance.

Suppose we find them wrong in some points, shall we set them down as fools? Shall we say, that because a "BELL" denied the vitality of dentine, therefore, Bell is no authority! For one I look up to them. I love their examples, and cherish their memories. I have made these few remarks to establish my position as it regards the popular *ipse dixit* of the profession. I differ from them in regard to the operation named at the head of this article. But it is an honest difference of opinion, and I wish merely to state a few facts and reasons for the difference.

It is well known that both in the writings and practice of the profession, the entire removal of fangs, and even scattering teeth is advised before inserting artificial pieces, and the contrary practice universally deprecated, so far as I am informed as to the public opinion of the profession.

Such being the case, I feel almost sure that in the next ten lines of this article, I shall be set down in the minds of some of my worthy brethren for the time at least as almost outside the pale of that profession I love, and whose honor I have pledged the labor of a life to sustain untarnished.

But to proceed. I have been, and am practically, theoretically, and conscientiously, in favor of setting teeth over fangs or roots of decayed teeth.

Do not be startled dear reader, and say hard things, but as you have read something that sounds as ominous to your ears, as a sentence of expulsion from a high and noble profession, let me ask two things as a matter of right. First, that you read carefully my defence, and secondly, that you suspend your judgment until you have put in twenty artificial pieces on plate keeping in mind my article. Depend upon it, you will be with me despite your legitimate prejudices. To be bold, I expect to convince you. One word before proceeding. I hold as sacredly as the truths my mother first taught me, *That no dentist has a right to swerve, for any cause, whatever, from what he considers the best interests of his patients. That they will not submit to this and that operation is no part of an argument, why he should vary his operations in the least.*

I have saved a dear sister, for ten years, the fangs of the incisors cuspidati, and two bicuspides, as a solid foundation for mastication with the alveolar process, and muscles of the face retained in their normal condition and use.

All my patients are as thoroughly my brothers or sisters, and what I dare not do for the latter, I dare not do for the first named. But to facts. The alveolar process has a use beyond the mere investment of the fangs of the teeth. Over this process is drawn another investment arranged with muscles, giving beauty and symetry, to the "face divine." Remove from this process the teeth, and it is absorbed, the muscles of the face are contracted or thrown out of use, and the youthful smile that was treasured up in the hearts of the happy mother, or husband, becomes a thing "dwelling greenly in their memories" only, for it no longer plays on the loved one's face. Disease has been there, and the dentist has been there, and between the two has stood, that shrouded monitor before his time to tell of the first fruits of mortality.

But here comes my patient. Stay brother dentist, and let us consult in the case, a word as to who she is. In that reception room is a daughter of the "sunny south." At home she is surrounded not only by all that wealth can procure, but she is the only child that gladdened the heart of that angel mother. But now, as that old man totters down the hill of life, he sees in his child the perfect youthful image of that lost one, the companion of his youth, and she lives again. She has also other friends. All love her. But there is one who in a few months will lead her to the altar, the cynosure of a hundred hearts.

But I will call her in. See! nature formed her beautiful, but those rosy lips disclose not pearls beneath. Let us examine. In the lower jaw she has well developed incisors, cuspldati, and bicuspides. The first molar is gone, the second is a pretty good tooth. The dens sapientiæ are just coming through, and have room to develop well. Above, the molars are in about the same condition. But the incisors, cuspidati, and bicuspides, have no crowns, that are susceptible of being filled, with the least promise of success or use. What shall be done?

See! That old father has her likeness in his hand, and as he looks at those classic features, those thin lips, and aqualine nose, he sighs to think that this is the last time that the original will resemble the picture, for he has brought her to have those ten upper teeth, front of the molars extracted, for he has been told by the physician, and all the dentists who have operated for her, that "*they must come out*," that there is no alternative. But then she can have such nice artificial ones ("*and the dentist can make them so easily*"). "Her aunt wears them, her cousins wear them," and if their faces are sharp, and the shape of the nose has lost that full nostril by the deep depression at the side, still the change has been so gradual, that no one speaks about it, and they being the same good creatures few persons ever suspect that their teeth are false. That picture in the father's hand was taken to day, and is a good one. But look through my longnette, and I will show you her picture, as it will appear six months hence, (if those teeth are removed). But you do not speak! you feel sad at the sight. Now *can our profession do nothing to save her friends that sight.* You say you are afraid if those fangs are not removed they will ulcerate and become diseased. True. But I have a doubt about that, and I intend giving her the benefit of that doubt.

Now I intend to clean out those fangs, by removing the pulp or its remains, and then fill those fangs to the very point, and I expect, in a few days to see those gums assume a healthy appearance, by a restoration to health, of the periostium (external) of the fang.

When I see this I intend to cut down those shattered crowns carefully to the point of juncture with the gum, taking care not to cut beyond that point, so as to wound the periostium if possible. Then after finishing my fillings to that shape, finish by fitting a plate with perfect bearings on the end of the fangs, on which I will adjust teeth, fitting "the festoon of gum" like a pivot tooth, securing by any method thought best in the case.

Time alone will tell whether I am right. You will admit two things. First that she will have the *most natural* set of artificial teeth that can be put in by any method. Secondly, that she will have also *the most useful* artificial piece while they last. If my adjustment has been perfect, *what will be the effect on those fangs as to their connection with the process or sockets?* You know well the effect of bringing into use in mastication even an unhealthy fang. Perhaps you will be surprised to find that with very slight alterations, (the work of an hour,) ten years hence those fangs appear about as healthy, and the piece remains about as useful, as when first adjusted. But such is the fact in some cases, which I could show you. But suppose the worst, suppose with all the alterations the fangs have to be removed in, I will say five years, (it being about the average of my most unfortunate operations of this class). The fact then stands thus, she has had the most useful set she will ever have, and for that period, (*five years*). Her mouth, gums, and the muscles of the face have been preserved in their normal shape and use, and she now at the end of five years, starts where she would have done five years before, had those teeth been then extracted, with only this difference, that the remaining teeth have not suffered half the abrasion, that five years would have caused, if the teeth had been placed on the *soft base* of gum, instead of the *firm one* of fangs, attach which way you please. In explaining this to an old and eminently successful dentist probably one of the first who filled fangs or roots of teeth. He said, "I consider it criminal for a dentist to extract any tooth that can be by any operation retained," and I go farther and say, I consider it *mal practice* to extract even fangs that by any operation and treatment can be retained. I have seen an upper molar tooth, that seventeen years ago was cut down to the gum, and the fangs filled, and I had rather be excused from the operation of extracting them even now, both as to the force required to dislodge them, and the loss of self respect by mal practice.

I owe to the practice of putting teeth over fangs the first practical lessons in fang filling; I have prepared fangs by filling in this way for eleven years. I dared not put them in without removing all tenderness from the nerve, and have filled up the nerve channel, as a means of purity only, but thus I blundered on what is now, I believe, considered correct practice, as to the extirpation of the pulps, and substituting a filling of gold.

I will offer no speculations as to the effect of cutting off the artery at the point of the fang, by the compression of a filling stoping that foramen. Neither will I offer theories as to the heightened or recuperating influence of the periostium,

(external) of the fang. But I will state as a fact, that I do not know that the effect of removing the pulp, and placing in the fang a perfectly impervious filling is usually followed by a restoration of health to the periostium of the fang, and that a tooth thus treated will improve in life-like appearance for a year after such operation. And I think it fair to conclude that it partakes of that wise arrangement of the animal economy by which one organ of the body is stimulated to unusual strength by the loss of a partner to the same tissue or organization.

Why is it that we sometimes see a pivot tooth, that has been carefully set last ten, and even fifteen years. In examining those cases, in resetting them, I have found that the pulp or its remains had been carefully removed; whereas, in most cases that have come under my eye, the fang was rapidly decaying, and I have been pretty sure to find the remains of the old dead pulp above the pivot, surely there is a well marked indication here.

It may not be known to all of the readers of the Register, but I think a fact that in all cases where a tooth with a dead nerve does not ulcerate, but is retained as we sometimes see them in a comparatively healty state, the foramen at the point of the fang is *hermetically sealed* by *nature herself*. Surely here is a fact also of value, indicating the operation of fang filling.

I have thus stated in outline my principles of practice in treatment of fangs, but will in a future article speak of *cases*, and endeavor to place this subject more plainly before the profession, but there is one point that I conceive greatly misunderstood as to inducement by way of interest in operating in the way proposed here. I make the assertion with the full light of facts that it requires more sacrifice of interest, and a more unswerving adherence to what is considered right, maintained against the wishes of patients, and the advice of physicians, than the popular mode of operating. I can point to numerous cases within the last year in my practice where I

have lost more than one thousand dollars worth of artificial work, because no money could induce me to extract "those ugly teeth, and put pretty ones in their place." Teeth that I believe can be so filled and treated, as to be retained with comfort and use, or fangs that I believe can be made serviceable by the same operation, I will not, *I dare not extract.*

